# Distribution of elements and their correlation in bran, polished rice, and whole grain

**DOI:** 10.1002/fsn3.1379

**Published:** 2020-01-09

**Authors:** Bao‐Min Yao, Peng Chen, Guo‐Xin Sun

**Affiliations:** ^1^ State Key Laboratory of Urban and Regional Ecology Research Center for Eco‐Environmental Sciences The Chinese Academy of Sciences Beijing China; ^2^ University of Chinese Academy of Sciences Beijing China

**Keywords:** bran, nutrient element, relationship, rice, toxic element

## Abstract

The relationship of toxic elements (As, Cd, Cr) and trace elements (Cu, Se, Ni, Zn, Mn) in rice bran and corresponding polished rice is not well known. A total of 446 rice grains were collected from paddy fields distributed across China, and the concentrations of 8 elements in rice bran and their corresponding polished rice were measured. The levels of As, Cd, Cr, and Se have a good linear relationship between rice bran and polished rice (*R* ^2^: .79, .97, .82, .99, respectively; all *p* < .001). Polishing rice could effectively remove the average contents of 44.4% As, 19.8% Cd, and 15.4% Cr in the whole grain, but caused the substantial losses of more than half of Mn and Ni (57.7% and 56.9%), and nearly one‐third (30.9%, 31.5%, and 29.1%) of Cu, Se, and Zn in brown rice although only about 10% of rice bran was milled. The "L" type correlation exists not only between As and Cd, but also between the nutrients Se, Mn, Ni, and the toxic elements As, Cd. These results indicated that As accumulation in rice could reduce the levels of essential mineral nutrients Mn, Ni, and Se. On the contrary, improving nutrient elements by fertilization could decrease the accumulation of some toxic elements. This provides a practical new idea for the prevention and control of rice As or Cd, and concomitantly improves the deficiency of nutrient elements in rice.

## Graphical abstract

Arsenic accumulation in rice reduced the levels of essential mineral nutrients (Mn, Ni, and Se). Improving nutrient elements by fertilization could decrease the accumulation of some toxic elements.

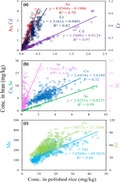

## 1 INTRODUCTION

Rice (*Oryza sativa* L.) is one of the most important food crops in the world as well as in China (Hu, Cheng, & Tao, 2016; Ray, Ramankutty, Mueller, West, & Foley, 2012). As the largest rice producer and consumer, (Chen et al., 2016; Ray et al., 2012) China has more than 65% of China's population with rice as staple food, and 18.3% of the total agricultural land for rice planting. The rice quality, including mineral nutrients and toxic elements, is closely related to the health of people who take rice as the main food. Cadmium (Cd) is one of the most harmful trace metals in rice and is considered to be the most serious contaminant in paddy fields (Shiraishi, 1975). It is a highly carcinogenic even at low concentrations (Honma et al., 2016; Meharg et al., 2013). Cd accumulates in the kidneys, liver, and other organs, (Band et al., 2008) causing diseases such as high blood pressure, osteoporosis, and kidney failure (Bernard, 2016; Haswell‐Elkins et al., 2007). Zhao et al. reported that rice is a major contributor of Cd to its consuming populations, with significantly higher contribution rate (71.1%) than that of other foods (Khan, Khan, Khan, Qamar, & Waqas, 2015; Zhao et al., 2017). Arsenic (As), as a carcinogen, is associated with many kinds of human diseases, including cardiovascular disease and neurological disorders (IARC, 2004). Many studies have shown that rice is the main pathway for human exposure to As, (Heikens, Panaullah, & Meharg, 2007; Meharg et al., 2009) because As concentration in rice is usually an order of magnitude higher than other cereal crops, (Williams et al., 2007) especially inorganic As (75.2%–96.5%) (Sun et al., 2008). Chromium (Cr) is a necessary trace element in human body, but if excessive intake, Cr would accumulate in the liver, kidney, and endocrine glands, posing a risk to human health. The pollution of As, Cd, and Cr in rice has caused seriously economic losses to China's agricultural production and brought serious health risks to local population (Hu et al., 2015; Yang, Wang, & Chen, 2017; Zhu, Chen, Xu, Zhu, & Huang, 2016; Zhu, Williams, & Meharg, 2008).

Copper (Cu), selenium (Se), nickel (Ni), zinc (Zn), manganese (Mn) are essential micronutrients in human diet, which are usually lacking in milled rice (FAOSTAT, 2007; GB2762‐2017; Tan et al., 2014). It is reported that about 2 billion population are suffering from the ‘hidden hunger’ worldwide due to micronutrient deficiency (Grebmer et al., 2014; He, Baiocchi, Hubacek, Feng, & Yu, 2018). More than five million childhood deaths occur from micronutrient malnutrition every year (Anonymous, 2007). Copper is the third most abundant trace metal in the body behind Fe and Zn with abundance of 0.08–0.1 g (Willis et al., 2005). Copper combines with protein or cytochrome oxidase to promote hematopoiesis, maintain the normal function of bone, blood vessels and skin, and protect the health of central nervous system and hair. Selenium is an important component of glutathione peroxidase and plays an important role in preventing tissue degeneration by acting as an antioxidant (Fordyce, Zhang, Green, & Xinping, 2000; Li et al., 2018; Xiao et al., 2018). In fact, 15% of the world's population is Se deficient (Combs, 2001). Deficiency of Se in human body would lead to a variety of diseases such as Keshan disease and Kashin‐Beck disease (Hartikainen, 2005). Zinc as second most prominent trace metal in human body after iron (Barceloux, 1999) is the only metal represented in all six enzyme classes, (Broadley, White, Hammond, Zelko, & Lux, 2007) and zinc deficiency seriously affects intelligence, appetite, and reproductive function (Sun et al., 2008). Ni is still an essential element in many mammalian species although the percentage of Ni lacks less than Se and Zn. In deprivation scenarios, it is considered to be a cause of stunting in addition to impairing the absorption of other micronutrients such as Zn and Cu (Huang, Gao, Wang, Staunton, & Wang, 2006). Manganese is related to the synthesis of mucopolysaccharide, an important component of cartilage and bone; and Mn deficiency leads to extensive skeletal deformity and easy fracture (Idouraine, Khan, & Weber, 1996).

In recent years, XRF (X‐ray fluorescence) and NanoSIMS techniques have been used to locate elements such as As, Se, Cu, Mn, and Zn in rice grains and the distribution of these elements in rice grains was mapped (Lombi et al., 2009; Moore et al., 2010). The concentration of As and micronutrients in rice bran were higher than that in corresponding endosperm (Sun et al., 2008; Williams, Lei, et al., 2009). However, the ratio of multielements in bran and corresponding polished rice is unclear. Although the relationship of individual element between rice bran and polished rice has been studied, there is no systematic investigation on the multiple relationships between toxic elements and nutrient elements in grain. The changes of element contents in polished rice and rice bran and their correlation with each other are of more theoretical and practical significance.

In this study, the concentrations of 3 toxic elements (As, Cd, and Cr) and 5 nutrient elements (Cu, Se, Ni, Zn, and Mn) in polished rice and corresponding rice bran were determined in rice samples (total 446 samples) from the main rice‐producing areas in China. The relationship of contents of individual elements in polished rice and rice bran was discussed. The correlation among different elements in rice bran and polished rice was discussed, which would provide valuable information for decreasing the concentrations of toxic elements by increasing the levels of nutrient elements.

## 2 MATERIALS AND METHODS

### 2.1 Sample collection and preparation

**Figure 1:**
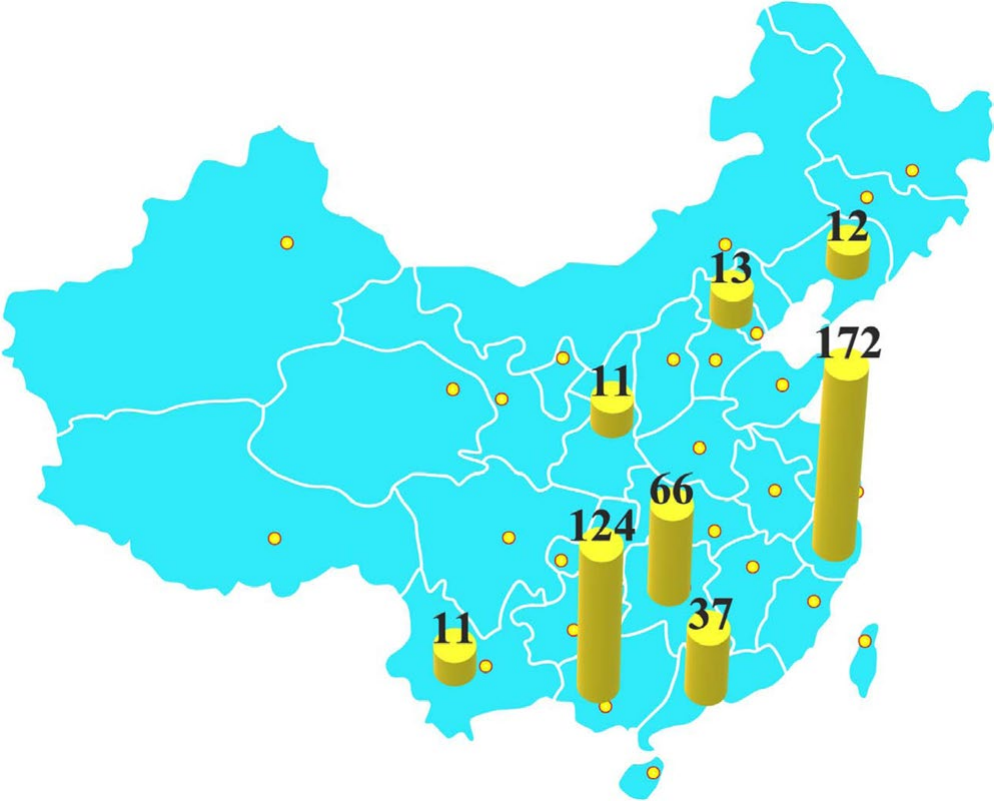
Distribution of rice sampling sites

In this study, a total of 446 rice grain samples were collected from paddy fields across main rice production area of China (Figure 1). Sample preparation was in accordance with our previous procedure (Sun et al., 2008). The rice grain is naturally air‐dried to a constant weight. All rice grains were dehusked with a motorized dehusker (JLGJ4.5, TZYQ), and subsamples of whole grains were milled using rice polisher (JNMJ3, TZYQ). The rice polishing process was as follows: Each brown rice (18.5 g) was weighed and put into a polisher and then polished for 90 s, during which the mass of rice bran and polished rice was weighed, and the quality of the ~10% was removed to obtain rice bran and polished rice. The whole grain and endosperm (polished rice) were then ground to a fine powder using a Moulinex Optiblend mixer. Rice bran is already in powder form.

### 2.2 Element concentration

The determination of the element concentrations in rice samples is based on our previous methodologies as described by Sun et al. (2008). Due to the large amount of samples, one out of every ten samples was randomly selected for triplicate. Each sample (0.2 g) was accurately weighed and put into a 50‐ml polyethylene centrifuge tube, and then added 2 ml of concentrated nitric acid. The tubes were stood for overnight and then subjected to microwave digestion (MARS, Matthew Inc.). The digestion program for microwaver is as follows. The temperature was ramped to 55°C over a 5 min and kept at 55°C for 5 min, then continuously ramped to 75°C over a 5 min and maintained at 75°C for 5 min, and then ramped to 95°C over a 5 min maintained for 30 min. After the digestion, the centrifuge tubes were placed in a fume hood until cooling. Each sample was diluted to 30 ml with Millipore ultrapure water (18.2 MΩ), then shaken by hand and kept in refrigerator at 4°C until analysis. Each digestion batch was accompanied by blanks and standard reference material (GBW(E) 100,357 Chinese rice flour). The element concentrations in each sample were analyzed using inductively coupled plasma mass spectrometry 7,700 (ICP‐MS, Agilent Technologies). The 7,700 ICP‐MS has an ORS collision/reaction cell, which can effectively eliminate the interference of particles such as Cl and Na on As, Cu, Cr, Zn, and Se during measurement. For the element concentrations of certain samples observed to be below the limit of detection (LOD: As, 0.047 µg/L; Cd, 0.012 µg/L; Cr, 0.031 µg/L; Ni, 0.024 µg/L; Mn, 1.613 µg/L; Cu, 0.494 µg/L; Zn, 0.619 µg/L; Se, 0.024 µg/L), an arbitrary value of 50% LOD was used. Validation analysis was performed using a certified reference material, and average recovery rates are in the range from 88% to 105%.

## 3 RESULTS

### 3.1 Analysis of the contents of 8 elements in the grain

**Figure 2:**
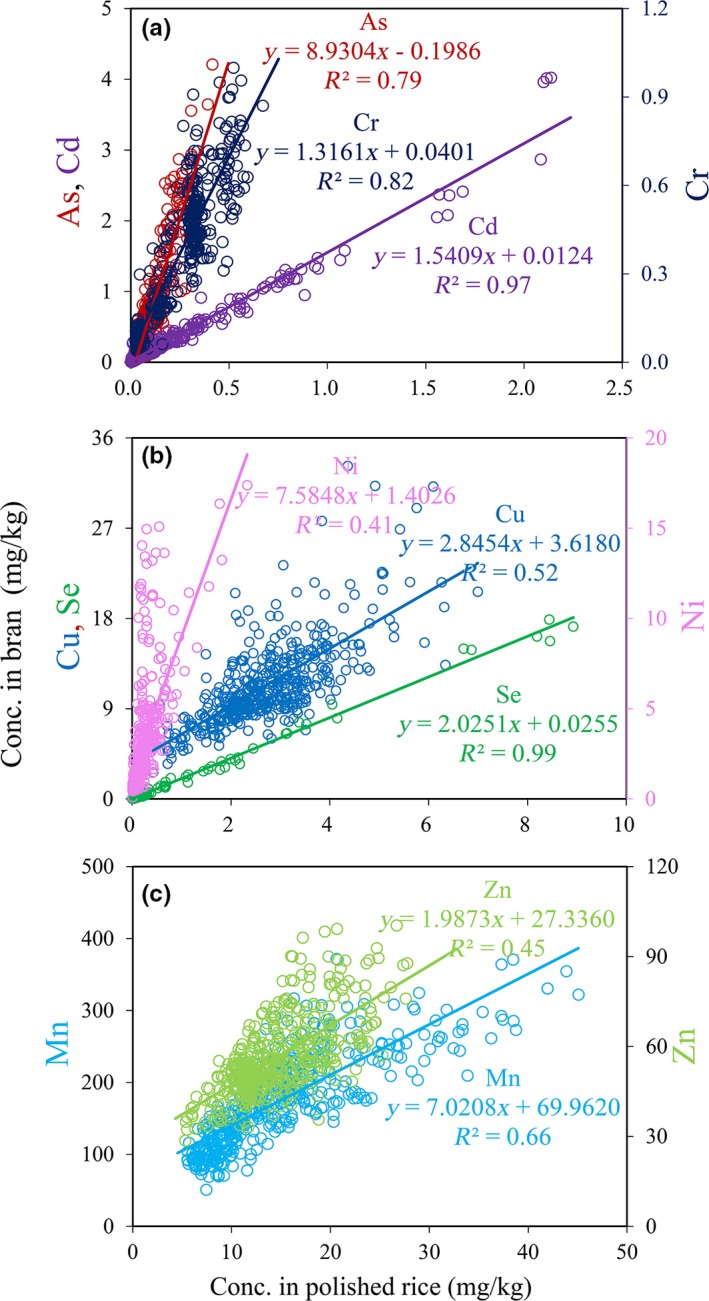
Histogram of distribution frequency of 8 elements in 446 samples under different concentration gradients in brown rice, rice bran and polished rice

The distribution frequency of 8 elements in 446 samples at different concentration ranges in brown rice, rice bran, and polished rice (Figure 2). The average concentrations of three toxic elements (As, Cd, and Cr) in whole grain were 0.196, 0.201, and 0.266 mg/kg, respectively (Table 1), and the corresponding ranges were 0.054–0.795, 0.002–2.326, and 0.033–0.691 mg/kg. It was reported that the As content in whole grain is 0.01–2.05 mg/kg in Bangladesh, 0.03–0.44 mg/kg in India, 0.10–0.76 mg/kg in Taiwan, 0.11–0.66 mg/kg in the United States, 0.03–0.47 mg/kg in Vietnam, and 0.08–0.38 mg/kg in Italy and Spain (Caroli, D’Ilio, Alessandrelli, Forte, & Caroli, 2002; Duxbury, Mayer, Lauren, & Hassan, 2003; Pizarro & Gómez, 2003; Williams et al., 2005). The Cd concentration in whole rice in southern China was 0.08–0.40 mg/kg (Yang, Lan, Wang, Zhuang, & Shu, 2006). Rice bran exhibited much higher concentrations (As, Cd, and Cr) on average of 0.842, 0.297, and 0.351 mg/kg, respectively (Table 1), and the corresponding maximum values were 4.208, 4.021, and 0.999 mg/kg. Both bran As and Cd levels are significantly higher than China's rice standard, which are 0.2 mg/kg inorganic As and 0.2 mg/kg Cd (GB2762‐2017) (Table 1 and Figure 2). All As concentrations in bran are above the China rice limit, and all Cr concentrations are below rice standard of 1.0 mg/kg Cr (Table 1 and Figure 2). The average concentrations of three toxic elements (As, Cd, and Cr) in polished rice were 0.117, 0.186, and 0.236 mg/kg with the corresponding maximum levels of 0.415, 2.138, 0.671 mg/kg. Ren et al. reported that As concentrations in China were the highest in bran (in the range of 0.55–1.20 mg/kg), followed by whole grain (0.14–0.80 mg/kg) and polished rice (0.07–0.4 mg/kg) (Ren, Liu, Wu, & Shu, 2006). The concentrations of all three toxic elements in bran were significantly higher than endosperm (polished rice). The average ratios of As, Cd, and Cr in rice bran and polished rice were 7.1, 2.2, and 1.7. These results suggested that As is mostly accumulated in rice bran, followed by Cd and Cr. By polishing rice, the average of 44.4% As, 19.8% Cd, and 15.4% Cr contents in whole grain could be removed, a bit higher than other reports. The average 38% As and 12% Cd in the whole grain was removed by polishing rice with the removal of ~10% rice bran (Gyuhan & Todor, 2019; Williams, Lei, et al., 2009).

**Table 1:** The average, median, and range contents of 8 elements in rice bran, polished rice, and bran/polished rice ratio (ICP‐MS)

	Whole rice (mg/kg)			Rice bran (mg/kg)			Polished rice (mg/kg)			Bran/polished rice ratio			Element contents loss (%)		
	Average	Median	Min‐max	Average	Median	Min‐max	Average	Median	Min‐max	Average	Median	Min‐max	Average	Median	Min‐max
As	0.196	0.158	0.054–0.795	0.842	0.628	0.082–4.208	0.117	0.098	0.032–0.415	7.1	6.7	1.1–16.0	44.4	44	10.5–75.5
Cd	0.201	0.076	0.002–2.326	0.297	0.117	0.003–4.021	0.186	0.069	0.001–2.138	2.2	1.7	1.0–11.0	19.8	17.2	3.9–55.1
Cr	0.266	0.307	0.033–0.691	0.351	0.374	0.039–0.999	0.236	0.284	0.025–0.671	1.7	1.5	0.5–4.5	15.4	14.4	5.7–33.3
Ni	0.529	0.364	0.020–3.836	3.33	2.457	0.103–17.4	0.26	0.198	0.009–2.330	18	12.5	2.6–78.0	56.9	55.6	12.9–89.2
Mn	31.0	29.5	12.8–74.9	174.3	168.5	51.0–371.8	14.9	13.3	5.5–45.1	12.5	12.6	6.2–20.5	57.7	58.8	20.5–75.4
Cu	3.560	3.409	0.540–8.600	11.4	10.5	2.665–33.2	2.743	2.634	0.426–6.993	4.4	4.2	2.0–10.2	30.9	31	4.4–53.2
Zn	19.0	18.2	7.648–34.1	56.4	53.8	28.6–100.4	14.6	13.9	5.3–27.8	4.0	4.0	1.9–6.7	31.5	30.9	13.8–49.7
Se	0.351	0.095	0.004–9.754	0.651	0.161	0.022–17.9	0.309	0.074	0.002–8.927	2.9	2.3	1.0–13.6	29.1	22.6	2.4–69.0

The average levels of five nutrients (Ni, Mn, Cu, Zn, and Se) in whole grain were 0.529, 31.0, 3.560, 19.0, and 0.351 mg/kg, respectively (Table 1). The average contents of these elements in rice bran were 3.330, 174.3, 11.4, 56.4, and 0.651 mg/kg, respectively (Table 1), significantly higher than the corresponding polished rice. Large proportions of Ni and Mn were accumulated in rice bran, which reached 18.0 times and 12.5 times of the average in polished rice. The loss of Ni and Mn in whole grain through polishing process was the largest as well, with an average removal of 56.9% and 57.7%. The average ratios of Cu, Zn, and Se in rice bran/endosperm were 4.4, 4.0, and 2.9, and nearly one‐third of Cu, Zn, and Se (30.9%, 31.5%, and 29.1%) were removed from brown rice by polishing. All of these results indicate that rice bran has higher proportions of nutrient elements, especially Mn and Ni. Polishing caused the great losses of more than half of Mn and Ni, and nearly one‐third of Cu, Se, and Zn in brown rice although only about 10% of rice bran was polished. Hansen et al. reported that the loss of Zn concentration was in the range of 14%‐59% after 450 s of polishing (about 15% dry matter loss) (Hansen et al., 2012). Gyuhan and Todor (2019) removed the average of 67.1% Mn, 21.4% Cu, and 27.9% Zn in the whole grain with the removal 11% rice bran.

### 3.2 Elemental correlation analysis of rice bran and polished rice

**Figure 3:**
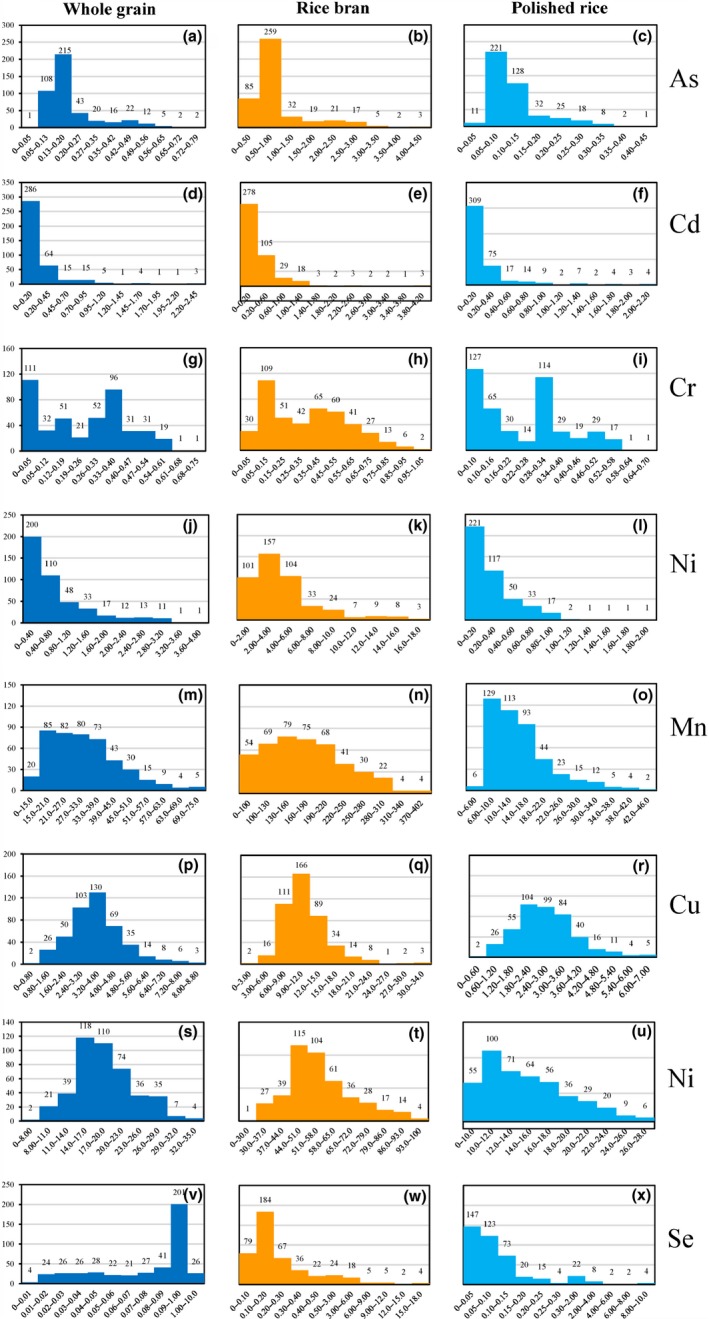
Relationship between Cd, As, Se, Cr, Mn, Cu, Zn, and Ni in rice bran and polished rice. All correlations were statistically significant (*p* < .001). *R* ^2^: (a) Cd, .97; As, .79; Cr, .82; (b) Cu, .52; Se, .99; Ni, .41; (c) Zn, .45; Mn, .66

We performed Pearson correlation analysis on the elements in rice bran and polished rice (Table 2). There are significant correlations for eight elements between rice bran and polished rice (all *p* < .001). The eight elements fit well (*R* ^2^: (A) Cd, .97; As, .79; Cr, .82; (B) Cu, .52; Se, .99; Ni, .41; (C) Zn, .45; Mn, .66), indicating that these elements have a significantly linear correlation between rice bran and polished rice (Table 2, Figure 3). The best linear relationship is Se (*R* ^2^: .99), and the worst one is Ni (*R* ^2^: .41) (Figure 3). There was a significant positive correlation between As and Cr (all *p* < .01; *R* ^2^: .426 in bran, .409 in polished rice) (Table 2). There was a significant negative correlation between As and Cd (*p* < .01; *R* ^2^: −.159 in polished rice), but not between Cd and Cr. The following correlations exist between the three toxic elements and the five nutrient elements: (a) very significant positive correlation (As & Cu, As & Zn, Cd & Ni, Cd & Cu, Cd & Zn, Cd & Se, all *p* < .01); (b) extremely significant negative correlation (As & Mn, *p* < .01, As & Ni, *p* < .01, As & Se, *p* < .05); and (c) Cr and 5 nutrients were extremely positively correlated at *p* < .01. Among the 5 nutrient elements, there are positive correlations among the four elements Ni, Mn, Cu, and Zn. Se is positively related to the other 3 nutrients (Ni, Cu, and Zn) except for the negative correlation with Mn (Table 2).

**Table 2:** Pearson correlation analysis of element contents in rice bran and polished rice

	BAs	BCd	BCr	BNi	BMn	BCu	BZn	BSe	RAs	RCd	RCr	RNi	RMn	RCu	RZn	RSe
BAs	1	0.092	0.000	0.001	0.000	0.000	0.000	0.098	0.000	0.025	0.000	0.001	0.000	0.000	0.542	0.045
BCd	−0.086	1	0.847	0.000	1.000	0.000	0.001	0.000	0.012	0.000	0.079	0.000	0.340	0.000	0.129	0.000
BCr	0.426**	−0.010	1	0.677	0.000	0.000	0.000	0.000	0.000	0.817	0.000	0.000	0.000	0.000	0.000	0.001
BNi	−0.178**	0.196**	−0.022	1	0.364	0.031	0.015	0.145	0.001	0.000	0.183	0.000	0.016	0.005	0.011	0.102
BMn	−0.299**	0.000	0.178**	0.047	1	0.101	0.011	0.081	0.000	0.657	0.000	0.000	0.000	0.000	0.000	0.111
BCu	0.300**	0.302**	0.453**	0.111*	0.080	1	0.000	0.216	0.000	0.000	0.000	0.000	0.719	0.000	0.000	0.295
BZn	0.190**	0.166**	0.506**	0.125*	0.125*	0.335**	1	0.000	0.070	0.001	0.000	0.000	0.461	0.000	0.000	0.000
BSe	−0.082	0.209**	0.175**	0.076	−0.086	0.060	0.248**	1	0.042	0.000	0.013	0.000	0.029	0.436	0.045	0.000
RAs	0.890**	−0.128*	0.362**	−0.169**	−0.302**	0.192**	0.088	−0.100*	1	0.002	0.000	0.008	0.000	0.022	0.817	0.022
RCd	−0.115*	0.983**	−0.012	0.224**	0.023	0.257**	0.175**	0.237**	−0.159**	1	0.074	0.000	0.648	0.000	0.042	0.000
RCr	0.491**	−0.091	0.905**	−0.069	0.186**	0.430**	0.457**	0.124*	0.409**	−0.092	1	0.000	0.000	0.000	0.000	0.016
RNi	−0.164**	0.278**	0.249**	0.642**	0.195**	0.207**	0.269**	0.189**	−0.136**	0.338**	0.201**	1	0.000	0.000	0.000	0.000
RMn	−0.301**	−0.049	0.219**	0.125*	0.812**	0.018	0.036	−0.107*	−0.227**	−0.023	0.210**	0.259**	1	0.000	0.000	0.049
RCu	0.186**	0.272**	0.418**	0.142**	0.218**	0.719**	0.261**	0.038	0.112*	0.258**	0.442**	0.352**	0.276**	1	0.000	0.454
RZn	0.030	0.078	0.508**	0.131*	0.319**	0.196**	0.673**	0.099*	0.011	0.104*	0.509**	0.338**	0.468**	0.459**	1	0.035
RSe	−0.099*	0.220**	0.172**	0.085	−0.079	0.051	0.244**	0.996**	−0.113*	0.252**	0.120*	0.204**	−0.097*	0.037	0.104*	1

Note

BAs and PAs represent the arsenic content in rice bran and the arsenic content in polished rice, respectively, and the other elements are also represented by the same method. The upper right of the table is the P value, and the lower left is the R value.

** Correlation is significant at the 0.01 level (2‐tailed).

* Correlation is significant at the 0.05 level (2‐tailed).

**Figure 4:**
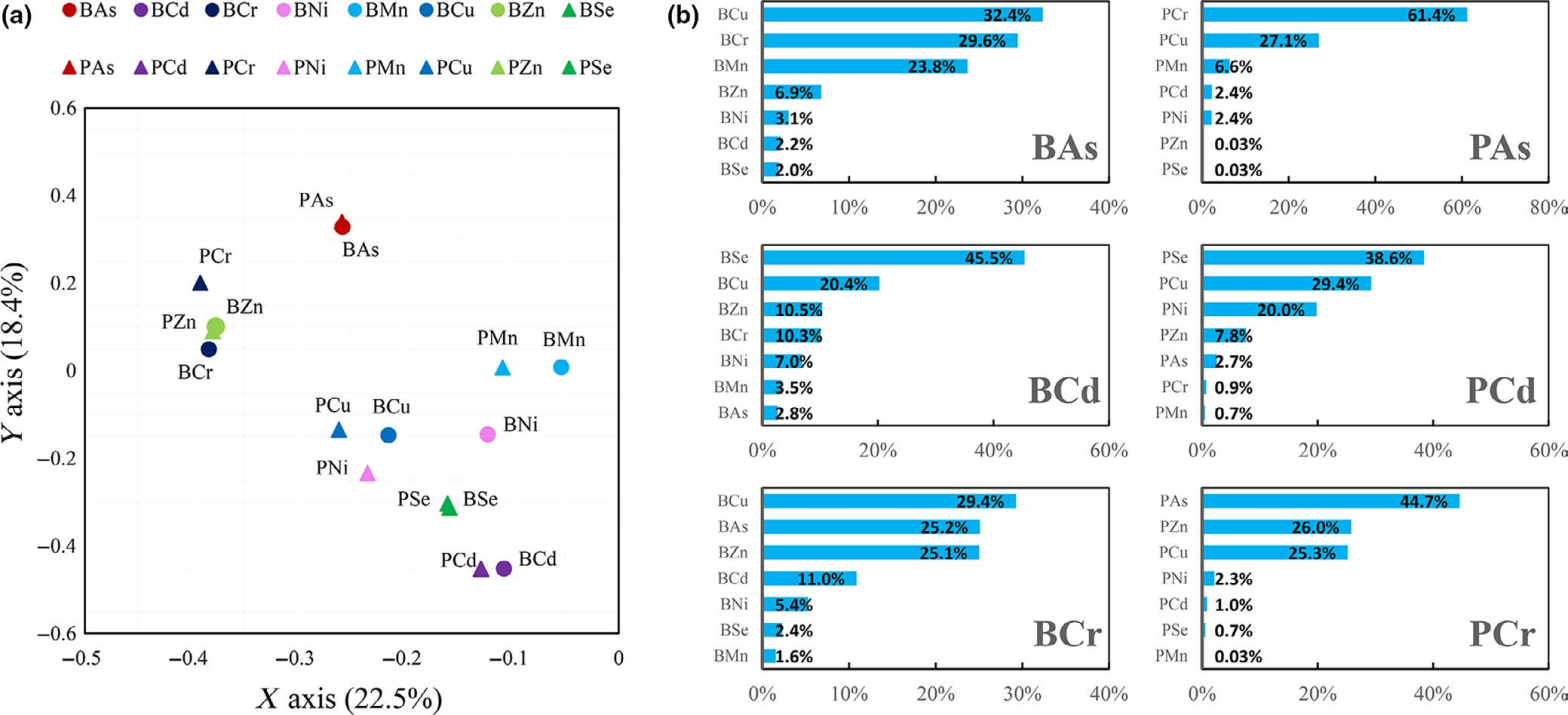
(a) Principal component analysis (PCA) of eight elements in rice bran and polished rice; (b) aggregated boosted tree (ABT) analysis of three toxic elements (As, Cd, Cr). BAs and PAs represent the As content in rice bran and polished rice, respectively, and the other elements are also represented by the same method

Principal component analysis (PCA) of the elements in rice bran and polished rice showed the closest distance between the same elements (except Cr and Ni) (Figure 4a). The three elements (As, Cr, and Zn) are closely clustered together, while the five elements (Cd, Ni, Mn, Cu, and Se) are more closely aggregated. Aggregated boosted tree (ABT) analysis showed that BCu (32.4%), BCr (29.6%), and BMn (23.8%) were the main factors of BAs, explaining the variation of BAs up to 85.8%, while PCr in polished rice was the most important factor of PAs, explained the variation up to 61.4%. And the ABT analysis (Figure 4b) of Cr also showed that there was a very high mutual interpretation percentage between As, Cr, and Cu. The Pearson correlation analysis in Table 2 also confirmed the strong correlation between these three elements (all *p* < .05). For Cd, Se is the most important factor both in rice bran and in polished rice, explaining the variation in BCd and PCd of 45.5% and 38.6%, respectively, which is supported by strong correlation between Cd and Se (Table 2, Figure 4).

**Figure 5:**
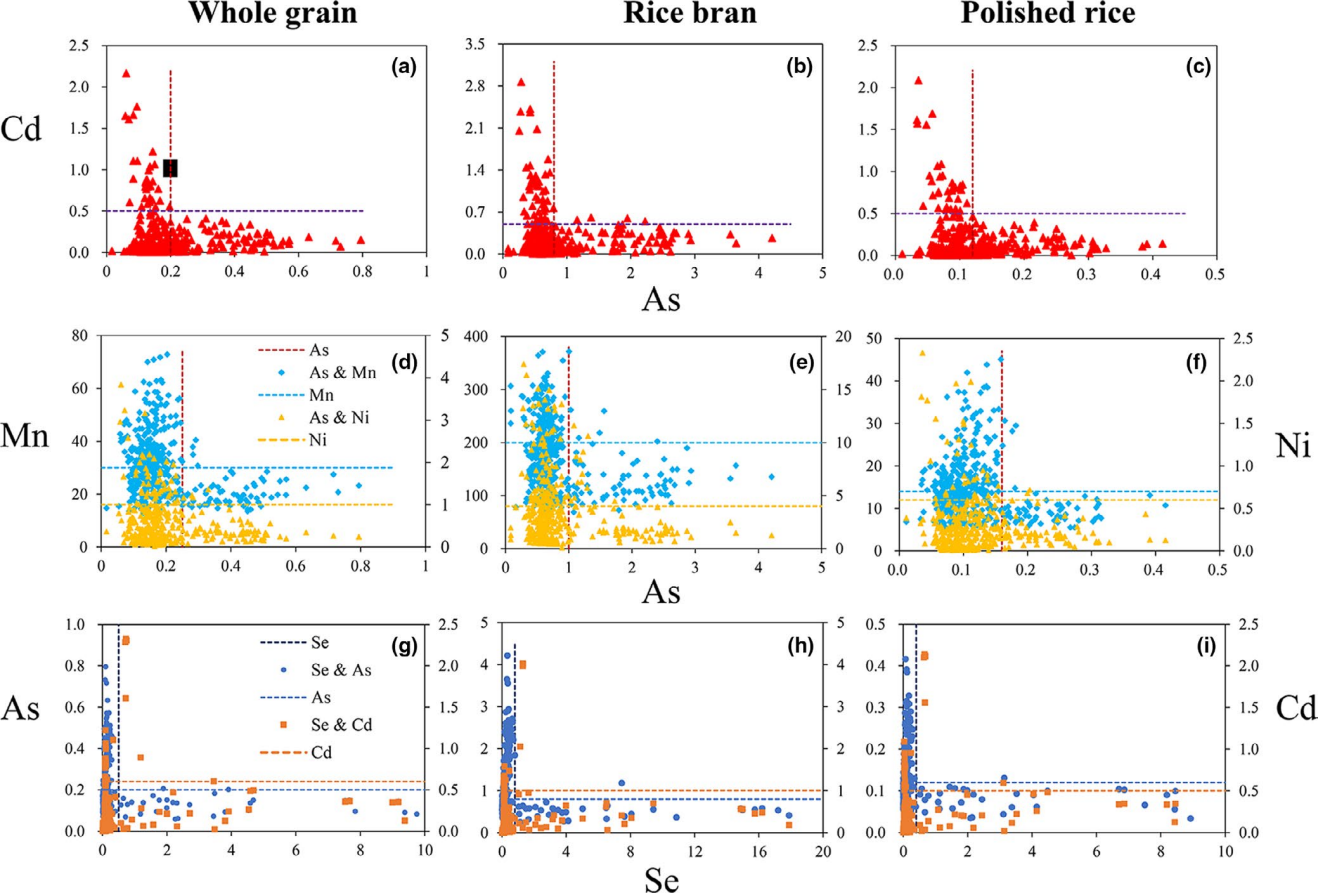
The relationship between As versus Cd; As versus Mn, Ni and Se versus As, Cd in whole grain, rice bran and polished rice

Some correlation among toxic elements and mineral elements could be described as the "L" type (Figure 5). That is, when the concentration of one element exceeds a certain threshold value, the enrichment of the other elements in rice would rapidly drop to a low level. For example, in Figure 5a–c, when the Cd concentration exceeds 0.5 mg/kg in whole grain, rice bran, and polished rice, the corresponding concentrations of As drop sharply to 0.2, 0.8, and 0.12 mg/kg. When the As concentration in whole grain, rice bran, and polished rice was higher than 0.2, 0.8, and 0.12 mg/kg respectively, the content of Cd decreased significantly (<0.5 mg/kg). Similarly, the relationship of As versus Mn & Ni (Figure 5d–f) showed that when the As concentration exceeded 0.25, 1.0, and 0.16 mg/kg, in whole grain, rice bran, and polished rice, the Mn or Ni levels in rice were decreased. When the concentrations of Mn in whole grain, rice bran, polished rice exceeded 30, 200, 14 mg/kg, or Ni level exceeded 1.0, 4.0, 0.6 mg/kg, the accumulation of As was inhibited. The relationships of Se versus As & Cd (Figure 5g–i) also reflect similar trends. When the concentration of Se exceeded 0.5, 0.8, and 0.4 mg/kg, in whole rice, rice bran, and polished rice, the accumulation of As and Cd by rice was inhibited. When the concentration of As in whole rice, rice bran, polished rice exceeded 0.2, 0.8, 0.12 mg/kg; or Cd exceeded 0.6, 1.0, 0.5 mg/kg, in whole rice, rice bran, and polished rice, the accumulation of Se was inhibited. The accumulation of trace elements Mn, Ni, and Se was inhibited when the As concentration exceeded 0.25, 1, 0.16 mg/kg, in whole rice, rice bran, and polished rice, respectively. Williams, Islam, et al. (2009)) reported that the concentrations of Ni, Zn, and Se in rice grain significantly declined with the increase of As levels. Our study showed that some nutrient elements can also inhibit the absorption of toxic elements, which also has guiding significance for rice agronomic cultivation.

## 4 DISCUSSION

Rice bran is an important by‐product produced in the process of polishing whole grain. It is mainly composed of pericarp, aleurone layer, subaleurone layer, seed coat, nucellar layer, germ, and endosperm (Gul, Yousuf, Singh, Singh, & Wani, 2015). In terms of nutritional potency, rice bran only accounts for 6%–10% of the total weight of rice, (Saikia & Deka, 2011; Wang, Khir, Pan, & Yuan, 2017) but contains 64% of important nutrients in rice and more than 90% of essential nutrients for the human body (Yang, Wang, Song, Jin, & Zhu, 2004). As a result, rice bran is widely used as food additives or as a "primary health food" (Meharg, Sun, et al., 2008; Sun et al., 2008). Our research showed that the toxic elements such as As, Cd, and Cr are enriched in rice bran as well besides essential minerals. For example, As concentration in bran is 7.1 times higher than polished rice (Table 1). Ren et al. observed that there were sixfold in As concentrations between rice bran and milled rice for a range of rice from various locations in China (Ren et al., 2006). It can be seen that nearly half As in whole grain (44.4%) were removed by polishing, similar to the other report (45%), (Norton et al., 2009) and the polished rice was suggested for consumption if whole rice grain contain high levels of As. Maximum food standard limit in China for As in rice is 0.2 mg/kg inorganic As (FAOSTAT, 2007), and 16.3% of all whole grain (446) in this study (inorganic arsenic is calculated as 75% of the total arsenic content (Adomako, Williams, Deacon, & Meharg, 2011; Sun et al., 2008; Williams et al., 2005) were in excess of this food standard. The over‐standard rate of polished rice decreased to 3.1% via polishing. The effect of polishing on Cd and Cr removal is not significant (both <20%). The over‐standard rate reduced from 2.8% (whole grain) to 2.7% (polished rice) for Cd.

High bran/polished rice ratio of As, Ni, and Mn (Table 1 and Figure 3) indicated that these three elements are mainly located in rice bran in comparison with polished rice. It is reported that As and Mn are mainly localized in the outer regions of the rice grains (i.e., aleurone/pericarp or outer parts of the endosperm), (Meharg, Sun, et al., 2008) and the areas where As and Mn mainly accumulation corresponds to the position of the ovular vascular trace (Hansen et al., 2012; Lombi et al., 2009). Lombi et al. believed that the larger concentration present in the bran compared with the corresponding polished rice may have two possible reasons. First, there could be a physiological barrier in the unloading and uploading process responsible for the transfer of As from the maternal tissues (ovular vascular system, either phloem, or xylem elements) to the filial tissues (aleurone). Second, As, as with many other elements, could accumulate preferentially in the protein‐rich aleurone and embryo tissues although (Lombi et al., 2009). This subaleurone region and vascular trace having high As level was removed by milling, resulting in marked decrease of As in polished rice and high As in bran. The distribution of Cd, Zn, and Cu is not limited to the aleurone/peel region, but gradually diffused into the inner layer, extends into the endosperm, and exhibits a relatively uniform distribution in the endosperm (Basnet, Amarasiriwardena, Wu, Fu, & Zhang, 2014; Gyuhan & Todor, 2019; Lombi et al., 2009; Meharg, Lombi, et al., 2008). The difference of elements distribution such as As and Cd in rice also explains that polishing brown rice has a better effect on removing As than Cd.

There is a very significant negative correlation between As and Ni or Mn in rice bran and between As and Ni, Mn, Se in polished rice (Table 2 and Figure 4). It has been reported that the increase of As content in rice declined the levels of trace minerals such as Se, Zn, Ni by perturbing grain metal(loid) balances, (Dwivedi et al., 2010; Norton et al., 2009; Williams, Islam, et al., 2009) constraining the concentrations of trace elements in the grain. The addition of inorganic Se suppressed the transfer of As from rice root to shoot in the hydroponic experiment (Hu, Duan, Huang, Liu, & Sun, 2014) and decreases As content in whole grain in pot experiment (Moulick, Santra, & Ghosh, 2018). Obviously, consumption of As‐contaminated rice not only post health risk from As directly, but also decrease the ingestion of microminerals via daily rice meal. Understanding the exact mechanism of specific element impacts on metal (loid) accumulation and distribution is rather difficult at present, considering many functional genes were involved. The situation is further complicated if the element species transformation were considered. However, we can improve rice qualities by applying micronutrient fertilizers regardless of the mechanisms. These fertilizations not only increases the levels of nutrient elements in rice grain, but also suppress the accumulation of toxic elements, and both are healthy for population.

In summary, our research shows that there is a good positively linear relationship of elements concentrations (including toxic and nutrient elements) between rice bran and milled rice. The highest ratio of bran versus polished rice is 18.0 for Mn, followed by 12.5 for Ni and 7.1 for As. These indicated that polishing caused loss more than half of Mn (56.9%) and Ni (57.7%), nearly half of As (44.4%) removal from grain. The negative relationships of element levels suggested that As contamination resulted in the mitigation of microminerals in rice grain. On the contrary, applying elements fertilizers could improve the contain of micronutrients as well as decrease As levels.

## CONFLICT OF INTEREST

The authors declare no conflict of interest.

## ETHICAL APPROVAL

This study does not involve any human or animal testing.
